# Hydroxysteroid 17β‐dehydrogenase 13 (*Hsd17b13*) knockdown attenuates liver steatosis in high‐fat diet obese mice

**DOI:** 10.1113/EP092535

**Published:** 2025-02-27

**Authors:** Shehroz Mahmood, Nicola Morrice, Dawn Thompson, Sara Milanizadeh, Sophie Wilson, Philip D. Whitfield, George D. Mcilroy, Justin J. Rochford, Nimesh Mody

**Affiliations:** ^1^ Aberdeen Cardiovascular & Diabetes Centre, School of Medicine, Medical Sciences & Nutrition University of Aberdeen Aberdeen UK; ^2^ Institute of Medical Sciences, School of Medicine, Medical Sciences & Nutrition University of Aberdeen Aberdeen UK; ^3^ Centre for Genome Enabled Biology & Medicine University of Aberdeen Aberdeen UK; ^4^ Glasgow Polyomics University of Glasgow Glasgow UK; ^5^ The Rowett Institute, School of Medicine, Medical Sciences & Nutrition University of Aberdeen Aberdeen UK

**Keywords:** fenretinide, fibrosis, hydroxysteroid 17β‐dehydrogenase 13 (Hsd17b13), metabolic dysfunction‐associated steatotic liver disease (MASLD), phospholipids

## Abstract

Hydroxysteroid 17β‐dehydrogenase 13 (HSD17B13) loss‐of‐function gene variants are associated with a decreased risk of metabolic dysfunction‐associated steatotic liver disease (MASLD). Our RNA‐seq analysis of steatotic liver from obese mice ± fenretinide treatment identified major beneficial effects of fenretinide on expression of hepatic genes including *Hsd17b13*. We sought to determine the relationship between *Hsd17b13* expression and MASLD and to validate it as a therapeutic target by liver‐specific knockdown. *Hsd17b13* expression, which is unique to hepatocytes and associated with the lipid droplet, was elevated in multiple models of MASLD and normalised with the prevention of obesity and steatotic liver. Direct, liver‐specific, shRNA‐mediated knockdown of *Hsd17b13* (*shHsd17b13*) in high‐fat diet (HFD)‐obese mice, markedly improved hepatic steatosis with no effect on body weight, adiposity or glycaemia. *shHsd17b13* decreased elevated serum alanine aminotransferase (ALT), serum fibroblast growth factor 21 (FGF21) levels, and markers of liver fibrosis, for example, expression of *Timp2*. *shHsd17b13* knockdown in HFD‐obese mice and *Hsd17b13* overexpression in cells reciprocally regulated expression of lipid metabolism genes, for example, *Cd36*. Global lipidomic analysis of liver tissue revealed a major decrease in diacylglycerols (e.g. DAG 34:3) with *shHsd17b13* expression and an increase in phosphatidylcholines containing polyunsaturated fatty acids (PUFA) for example, phosphatidylcholine (PC) 34:3 and PC 42:10. Expression of key genes involved in phospholipid and PUFA metabolism, for example, *Cept1*, was also reciprocally regulated suggesting a potential mechanism of Hsd17b13 biological function and role in MASLD. In conclusion, *Hsd17b13* knockdown in HFD‐obese adult mice was able to alleviate MASLD via regulation of fatty acid and phospholipid metabolism, thereby confirming HSD17B13 as a genuine therapeutic target for MASLD and the development of liver fibrosis.

## Abstract (highlights)

- **What is the central question of this study?**
  Hydroxysteroid 17β‐dehydrogenase 13 (HSD17B13) loss‐of‐function gene variants are associated with a decreased risk of metabolic dysfunction‐associated steatotic liver disease (MASLD) and metabolic dysfunction‐associated steatohepatitis (MASH): what is the relationship between *Hsd17b13* expression and MASLD and can HSD17B13 be validated as a therapeutic target by liver‐specific knockdown?
- **What is the main finding and its importance?**
  Liver‐specific shRNA knockdown of *Hsd17b13* in obese mice markedly improved hepatic steatosis and markers of liver health, for example, serum alanine aminotransferase and fibroblast growth factor 21 levels. Hsd17b13 influenced the expression of lipid/phospholipid metabolism genes and phosphatidylcholines PC 34:3 and PC 42:10. Our study suggests a mechanism of Hsd17b13's biological function and a strong rationale behind targeting HSD17B13 for MASLD/MASH.

## 1 INTRODUCTION

Metabolic dysfunction‐associated steatotic liver disease (MASLD), previously termed non‐alcoholic fatty liver disease (NAFLD) is a common complication associated with obesity and diabetes. MASLD can progress to the more severe metabolic dysfunction‐associated steatohepatitis (MASH), previously termed non‐alcoholic steatohepatitis (NASH), and therefore requires urgent therapeutic attention (Pafili & Roden, 2021). MASLD is characterised by an accumulation of triglycerides in the liver and is estimated to affect 25–30% of the global population as well as up to 90% of patients classed as morbidly obese (Pafili & Roden, 2021). Other than dietary and lifestyle interventions, currently there is only one approved therapeutic available for MASLD/MASH (resmetirom, an oral selective thyroid hormone receptor (THR)‐β agonist), but human trials suggest medicines with known benefits against obesity and diabetes may be useful to also treat liver steatosis/steatohepatitis (Luo et al., 2022; Sookoian & Pirola, 2024). In addition, several novel pharmacological therapies are in various phases of clinical development for the specific treatment of MASLD/MASH (Esler & Bence, 2019).

Several studies including human genome‐wide association studies (GWAS) and multi‐omics data analysis have identified genes that are key drivers of MASLD (Chella Krishnan et al., 2018). For example, 17β‐hydroxysteroid dehydrogenase 13 (HSD17B13) is a novel lipid‐droplet protein that has been identified from human steatotic liver biopsies (Su et al., 2014). Moreover, several gene variants of *HSD17B13* linked to MASLD/MASH have been identified by GWAS performed in cohorts around the world (Amangurbanova et al., 2023). Three independent gene variants of *HSD17B13* which generate loss‐of‐function proteins are reported to be associated with protection from liver injury (Abul‐Husn et al., 2018; Chella Krishnan et al., 2018; Ma et al., 2019). HSD17B13 gene expression and protein levels are upregulated in patients and mouse models of MASLD and overexpression of *Hsd17b13* in mice promotes rapid lipid accumulation in the liver (Su et al., 2014). However, the traditional gene knockout of *Hsd17b13* in mice had no beneficial effect on the development of liver steatosis with various dietary interventions (Ma et al., 2021). This surprising result suggests that the normal biological role of HSD17B13 and its metabolic pathway may be important for normal liver function from birth, but abnormally elevated levels of HSD17B13 in adulthood are pathological drivers of the steatotic liver.

We have previously reported the beneficial effects of the synthetic retinoid fenretinide to inhibit adiposity, insulin resistance and the accumulation of liver triglycerides (Mcilroy et al., 2013; Morrice et al., 2017). In the present study, our analysis of unbiased, whole‐genome RNA‐sequencing in liver tissue from mice with high‐fat diet (HFD)‐induced obesity and type‐2 diabetes and mice treated with fenretinide to inhibit MASLD (Mcilroy et al., 2013; Morrice et al., 2017) revealed genes that are key drivers of MASLD including peroxisome proliferator‐activated receptor α (PPARα) targets, lipid‐droplet proteins (e.g. *Hsd17b13*), pro‐fibrotic genes and phospholipid metabolism genes. HSD17B13 has been reported to have dehydrogenase activity towards retinol and thus modulate retinoic acid (RA) levels (Ma et al., 2019). Thus, together with the human gene variant data on *HSD17B13* and liver disease, we were attracted to investigating this protein further. We hypothesised that direct inhibition of elevated levels of liver Hsd17b13 using RNAi‐mediated knockdown in vivo may reveal a beneficial phenotype and lead to novel insights into the biological function of this uncharacterised lipid‐droplet protein.

## 2 METHODS

### 2.1 Ethical approval

All animal procedures were performed under a project license approved by the UK Home Office under the Animals (Scientific Procedures) Act 1986 (PPL P1ECEB2B6) and the University of Aberdeen Ethics Review Board. Studies were performed following the recommendations in the ARRIVE guidelines under guidance by the Veterinary Surgeon and Animal Care and Welfare Officers of the institutional animal research facility.

### 2.2 Animal studies

The fenretinide HFD and *db*/*db* studies were previously described (Mcilroy et al., 2013; Morrice et al., 2017). For the *Hsd17b13* knockdown study, 3‐ to 4‐week‐old male C57BL/6J mice were purchased from Charles River, Edinburgh. Mice were maintained at 22°C–24°C on a 12‐h light–dark cycle with free access to food and water. At 11 weeks of age, 18 mice were placed on a 45% kcal HFD (Research Diets, NJ, D12451) whilst eight remained on regular chow. Following 21 weeks on HFD, mice were randomised into two groups for intraperitoneal (i.p.) injection of AAV8‐scrambled control (*shScrmbl*) or AAV8‐*shHsd17b13* at a virus titre of 1 × 10^11^ virus particles. Vector and shRNA information can be found at Vectorbuilder https://en.vectorbuilder.com id: VB180117‐1020znr and id: VB200302‐1251whe, respectively. Chow controls did not receive AAV8‐*shScrmbl* since these mice were an order of magnitude healthier in comparison to the 21‐week HFD‐induced obese mice with MASLD and thus receiving AAV8‐*shScrmbl* would not inform any useful findings. Chow mice received a saline injection of the same volume administered by i.p. injection. Body weight was measured weekly and physiological tests were performed. Two weeks (14 days) after virus‐shRNA injections, mice were humanely killed in accordance with the guidelines and regulations detailed above (following 5 h of fasting) by CO_2_‐induced anaesthesia and cervical dislocation. Trunk blood following decapitation was collected and serum was stored at −80°C. All tissues were rapidly dissected, frozen in liquid nitrogen, and stored at −80°C. Tissues for histology were fixed in formalin for 48 h at 4°C, then stored at 4°C in phosphate‐buffered saline (PBS) until further processing.

### 2.3 Physiological tests

Body composition was measured before virus‐shRNA injection to confirm HFD‐induced obesity and aid with randomisation into treatment/control groups. The measurement was repeated 7 days after shRNA treatment. Each mouse was analysed using an Echo MRI‐3‐in‐1 scanner (Echo Medical Systems, Houston, TX, USA). A glucose tolerance test (GTT) was performed at 10 days post‐shRNA treatment. Briefly, mice were fasted for 5 h, and baseline glucose levels were sampled from tail blood using glucose meters (AlphaTRAK, Abbott Laboratories, Abbot Park, IL, USA). Subsequently, mice were injected i.p. with 20% glucose (w/v) and blood glucose was measured at 15, 30, 60 and 90 min post‐injection.

### 2.4 Stable expression of HSD17B13 in cells

HEK293 cells and HepG2, were maintained in Dulbecco's modified Eagle's medium (DMEM) GlutaMAX with 10% fetal bovine serum (FBS) at 37°C and 5% CO_2_. Human *HSD17B13* (NCBI Accession number: NM_178135.5) with HA‐tag on the C‐terminus was cloned into pcDNA3.1 (Genscript). HEK293 cells and HepG2 cells were transfected with *HSD17B13* or empty control vector using Lipofectamine 2000, cultured for 2–3 weeks in G418 for neomycin resistance, and positive colonies selected. Stable cell lines were treated with RA (1 µm, 2 h) or dimethyl sulfoxide (DMSO, vehicle control). An unsaturated/saturated fatty acid mixture (2:1 ratio of 0.66 mM oleic acid and palmitic acid 0.33 mM, 24 h) or 1% methanol (vehicle control) was prepared with 10% bovine serum albumin (fatty acid free) and 1% FBS in DMEM+G418.

### 2.5 Immunoblotting

Frozen liver tissues were homogenised in 400 µL of ice‐cold radioimmunoprecipitation assay buffer (10 mM Tris–HCl pH 7.4, 150 mM NaCl, 5 mM EDTA pH 8.0, 1 mM NaF, 0.1% SDS, 1% Triton X‐100, 1% sodium deoxycholate with freshly added 1 mM NaVO_4_ and protease inhibitors) using a PowerGen 125 homogeniser, Fisherbrand, UK and lysates normalised to 1 µg/µL. Proteins were separated on a 4–12% Bis–Tris gel (Thermo Fisher Scientific, Waltham, MA, USA) by gel electrophoresis and transferred onto a nitrocellulose membrane. Membranes were probed with the following primary antibodies: HSD17B13 (1/500, ab122036, Abcam, Cambridge, UK), GFP (1/1000, A11122, Thermo Fisher Scientific), glyceraldehyde 3‐phosphate dehydrogenase (GAPDH) (1/1000, 14C10) and Vinculin (1/1000, 13901) both Cell Signaling Technology (Danvers, MA, USA). Immunoblotting for retinol‐binding protein (RBP) (Dako, Agilent, USA) was from 1 µL serum (Yang et al., 2005).

### 2.6 RNA extraction and RT‐qPCR

Frozen tissues were lysed in TRIzol reagent (Sigma‐Aldrich, Gillingham) and RNA was isolated using phenol/chloroform extraction according to the manufacturer's instructions. RNA was then synthesised into cDNA (Tetro kit, Bioline, UK) and subjected to qPCR analysis using SYBR green and LightCycler 480 (Roche, Burgess Hill, UK). Gene expression was determined relative to the reference gene *Nono* for mouse liver tissue and *Hprt* for human cell lines (HEK and HepG2) and analysis was performed using the Pfaffl method (Pfaffl, 2001). Primer sequences are available on request.

### 2.7 Liver histology

Liver tissues were embedded in paraffin, sectioned and stained with haematoxylin and eosin (H&E). Images were taken using a light microscope, EVOS XL (Thermo Fisher Scientific, Gillingham, UK) at ×20 and ×40 magnification. Frozen liver tissue was used for fluorescence staining. Briefly, processed slides were washed with PBS for 10–12 min. Subsequently, slides were incubated with Blocking (5% goat serum in PBS) and then with HCS Lipidtox deep red neutral lipid stain (Thermo Fisher Scientific, H34477) (1:200 in PBS) at room temperature for 1 h. Furthermore, they were mounted with fluoroshield mounting medium with 4′,6‐diamidino‐2‐phenylindole (DAPI) (Abcam, ab104139).

### 2.8 Liver and serum assays

A total of 50–100 mg of frozen liver tissues was homogenised in 1 mL of PBS and frozen in liquid nitrogen to enable further cell lysis. Samples were left to thaw at room temperature and centrifuged for 15 s at 7000 *g* to pellet debris. The remaining fat cake was resuspended in PBS, and the resulting supernatant was used to determine total liver triglycerides according to the manufacturer's instructions (Sigma‐Aldrich, cat: MAK266). Serum was analysed for alanine aminotransferase (ALT) activity (Abcam, ab105134), fibroblast growth factor 21 (FGF21) (R&D Systems, Minneapolis, MN, USA, cat. no. MF2100) and total triglycerides (Sigma‐Aldrich, cat. no. MAK266) following manufacturer's guidelines.

### 2.9 Lipidomics analysis

Global lipidomics analysis was performed by high resolution liquid chromatography–mass spectrometry (LC‐MS) using an Exactive Orbitrap mass spectrometer (Thermo Fisher Scientific) interfaced to a Thermo UltiMate 3000 RSLC system. Samples (10 µL) were injected onto a Thermo Hypersil Gold C18 column (1.9 µm; 2.1 mm × 100 mm) maintained at 50°C. Mobile phase A consisted of water containing 10 mM ammonium formate and 0.1% (v/v) formic acid. Mobile phase B consisted of a 90:10 mixture of isopropanol–acetonitrile containing 10 mM ammonium formate and 0.1% (v/v) formic acid. The initial conditions for analysis were 65% A–35% B, increasing to 65% B over 4 min and then to 100% B over 15 min, held for 2 min prior to re‐equilibration to the starting conditions over 6 min. The flow rate was 400 µL/min. Analyses were undertaken in positive and negative ion modes at a resolution of 100,000 over the mass‐to‐charge ratio (*m*/*z*) range of 250 to 2000. Progenesis QI v3.0 (Nonlinear Dynamics, Newcastle upon Tyne, UK) was used to process the data sets and lipids were identified through interrogation of HMDB (http://www.hmdb.ca/), LIPID MAPS (www.lipidmaps.org/). Multivariate statistical analysis was performed using SIMCA‐P v13.0 (Umetrics, Umea, Sweden) and the heatmap was generated with the online available platform MetaboAnalyst 5.0 (https://www.metaboanalyst.ca/) (Pang et al., 2021).

### 2.10 RNA sequencing

As previously described (Morrice et al., 2017), total RNA was extracted from frozen liver using TriZOL reagent according to the manufacturer's instructions. RNA was purified using an RNeasy miniprep kit (Qiagen, Manchester, UK) and quantified on a Bioanalyzer 2000 (Agilent Technologies, Edinburgh, UK). Ribosomal RNA was removed from samples using the Ribo‐Zero rRNA removal kit (Illumina, San Diego, CA, USA). Sequencing libraries were prepared using the TruSeq RNA Library Preparation Kit v2 (Illumina) following the low sample protocol, using 1 µg RNA. Libraries were sequenced on the NextSeq‐500 Desktop sequencer platform (Illumina) with a 2 × 75 paired‐end read length giving 719 million raw reads (average depth of 45 million reads per sample). Reads were filtered using the FASTQ Toolkit v2.0.0 in the Illumina BaseSpace cloud computing environment to remove adapter sequences and read with a phred score <20 or length <20 bp. Filtered reads were checked using FastQC before aligning with STAR 2.0 to the *Mus musculus* UCSC mm10 genome and differential expression was measured using DESeq2 with default parameters. RNA‐seq data generated in this study have been deposited in the NCBI Gene Expression Omnibus (GEO) database with accession number GSE220684.

### 2.11 Data and statistical analysis

All values are expressed as individual values with mean and standard deviation as per the journal requirements, unless otherwise stated, using GraphPad Prism software (GraphPad Software versions 6‐8, Boston, MA, USA). Where data points are too numerous (e.g. body weight and GTT time course data), the entire raw dataset is available as Supporting information. Statistical analyses were performed by using one‐ or two‐way ANOVA (where stated) followed by multiple‐comparison *post hoc* tests to compare the means of three or more groups. Exact *P*‐values for posthoc comparisons between groups are presented in all figures, up to *P* ≤0.001 using IBM SPSS Statistics software.

## 3 RESULTS

### 3.1 Hepatic RNA‐seq reveals signature gene expression alterations in HFD‐induced obese steatotic liver and rescue by beneficial effects of fenretinide

**FIGURE 1:**
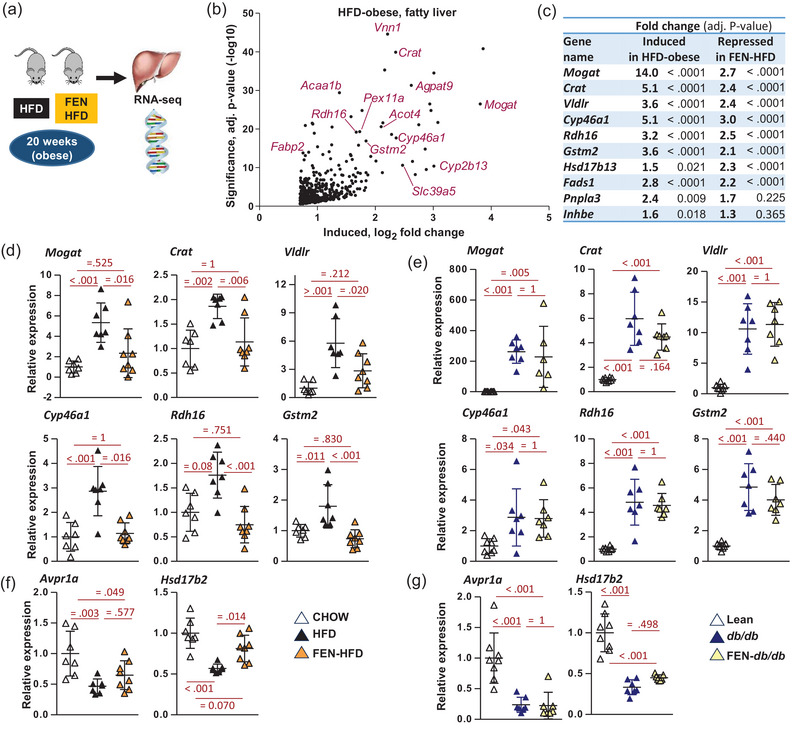
Hepatic gene expression in HFD‐induced obese and genetically obese mice with liver steatosis, and effects of fenretinide (FEN) treatment. (a) Graphical illustration of mouse liver study described. (b) Volcano plot of hepatic RNA‐seq data of HFD (obese‐steatotic liver) most highly induced genes by fold change and significance, compared to lean control mice (Morrice et al., 2017). (c) Shortlist of differential gene expression from RNA‐seq data, reciprocally regulated genes in HFD (obese‐steatotic liver) ± FEN, involved in triglyceride synthesis and fatty acid metabolism and genes associated with MASLD. HFD‐induced genes versus lean control mice and FEN‐HFD‐repressed genes versus HFD, *n* = 4 per group with mean fold change and exact adjusted *P*‐value. For further data, full RNA‐seq data have been deposited at NCBI GEO GSE220684. (d, f) Hepatic gene expression in HFD (obese‐steatotic liver) mice ± FEN. (e, g) Hepatic gene expression in *db/db* (genetically obese steatotic liver) mice ± FEN. Data (*n* = 7–8 per group) are presented as individual data points, means ± SD, and analysed by one‐way ANOVA followed by Bonferroni multiple comparison tests with exact *P*‐values for *post hoc* comparisons between groups (in red). HFD, high‐fat diet; MASLD, metabolic dysfunction‐associated steatotic liver disease.

We have previously reported the effects of fenretinide to inhibit adiposity, insulin resistance and the accumulation of liver triglycerides (Mcilroy et al., 2013; Morrice et al., 2017). In a novel approach to characterise the molecular mechanism of beneficial effects of fenretinide in the liver, we performed RNA‐seq (GEO number GSE220684) on liver tissue from HFD‐induced obese C57BL/6J mice ±fenretinide for 20 weeks, compared to lean control mice (Figure 1a) (Mcilroy et al., 2013; Morrice et al., 2017). HFD‐induced obesity resulted in the upregulation of 941 liver genes and the downregulation of 637 genes (Figure 1b). Many of the genes upregulated in HFD steatotic liver were involved in triglyceride synthesis and fatty acid metabolism and were well‐established targets of PPARα signalling and/or GGWAS‐identified genes associated with MASLD (e.g. *Mogat*, *Agpat9*, *Crat and Vnn1*, *Hsd17b13*, *Pnpla3* and *Inhbe*; Figure 1c) (Kersten, 2014). Fenretinide treatment partially prevented (and in some cases completely normalised) the increase in expression of many of these genes (Figure 1c).

HFD in the C57BL/6J strain is typically reported not to induce fibrosis or inflammation and therefore other diets are utilised to induce hepatic injury (e.g. Gubra Amylin NASH (GAN) diet or choline‐deficient HFD) (Loft et al., 2021; Vacca et al., 2024). However, our RNA‐seq revealed that HFD‐induced obesity in C57BL/6J mice for 20 weeks did induce increased expression of genes driving fibrosis and tissue remodelling, for example, collagens (*Col1a1*, *Col12a1* and *Col15a1*), tissue inhibitors of matrix metalloproteinases (*Timp1* and *Timp2*) and canonical Yap/Taz signalling pathway (*Wwtr1* and *Ctgf*) (GEO number GSE220684 and data available on request). Moreover, fenretinide treatment prevented the increase in expression of many of these genes.

To further characterise the effects on differential gene expression changes linked to steatotic liver, we compared the effects of fenretinide on hepatic gene expression in HFD‐induced and genetically obese *db/db* mouse models (Morrice et al., 2017). Both HFD and *db/db* obese mice had increased expression of hepatic lipid metabolism genes (e.g. *Mogat* and *Crat*) (Figure 1d, e). Fenretinide treatment prevented this increase in HFD mice but had no effect in *db/db* mice (Figure 1d, e). Similarly, HFD repressed other genes (e.g. *Avpr1a* and *Hsd17b2*) and fenretinide treatment prevented this decrease in HFD mice but had no effect in *db/db* mice (Figure 1f, g). These novel findings regarding the mechanism of fenretinide action suggest that the beneficial effects on hepatic gene expression are associated with inhibition of adiposity (induced by HFD feeding) and associated insulin resistance and MASLD but not with fenretinide treatment in genetically obese *db/db* where adiposity and MASLD were not inhibited by this synthetic retinoid (Mcilroy et al., 2013; Morrice et al., 2017).

### 3.2 *Hsd17b13* expression is upregulated in HFD‐induced and genetic mouse models of obesity, hyperglycaemia and MASLD

**FIGURE 2:**
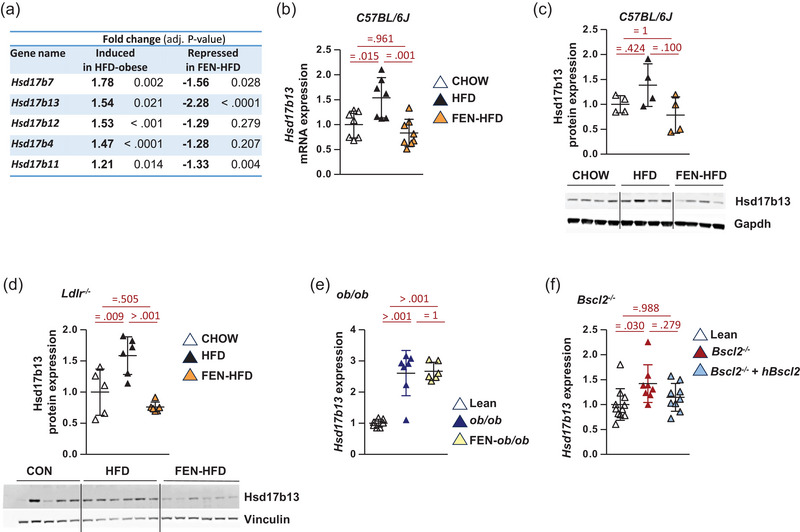
Increased *Hsd17b13* expression in an HFD‐induced and genetic mouse model of obesity, hyperglycaemia and MASLD. (a) Differential gene expression from RNA‐seq data. Hydroxysteroid 17β‐dehydrogenase family of genes reciprocally regulated in HFD (obese‐steatotic liver) ± FEN. HFD‐induced genes versus lean control mice and FEN‐HFD‐repressed genes versus HFD data, *n* = 4 per group with mean fold change and exact adjusted *P*‐value. For further data, full RNA‐seq data have been deposited at NCBI GEO GSE220684. (b) *Hsd17b13* gene expression in HFD (obese‐steatotic liver) mice ± FEN and chow‐fed lean controls, normalised to *Nono* (*n* = 7–8 per group). (c) Western blot of HSD17B13 protein expression in HFD (obese‐steatotic liver) mice ± FEN and chow‐fed lean controls, and quantification normalised to GAPDH (*n* = 4 per group). (d) Western blot and quantification of HSD17B13 protein expression in *Ldlr^−/−^* HFD (obese‐steatotic liver) mice ± FEN and control (10% kcal) fat diet lean controls, and quantification normalised to vinculin (*n* = 5–6 per group). (e) *Hsd17b13* gene expression in *ob/ob* (genetically obese steatotic liver) mice ± FEN and control (10% kcal) fat diet lean controls, normalised to *Nono* (*n* = 6–7 per group). (f) *Hsd17b13* gene expression in *Bscl2* ^−/−^ (lipodystrophic steatotic liver) mice, human *Bscl2* ^−/−^ rescue and wild‐type control, normalised to *Nono* (*n* = 8–9 per *Bscl2* ^−/−^ groups, *n* = 11 wild‐type control). Data are presented as individual data points, means ± SD, and analysed by one‐way ANOVA followed by Bonferroni multiple comparison tests with exact *P*‐values for *post hoc* comparisons between groups. HFD, high‐fat diet; MASLD, metabolic dysfunction‐associated steatotic liver disease.

17β‐Hydroxysteroid dehydrogenases (17β‐HSDs) are a family of 15 members that play a key role in sex hormone metabolism by catalysing steps of steroid biosynthesis (e.g. Hsd17b1 and b2) (Marchais‐Oberwinkler et al., 2011; Poutanen & Penning, 2019). Some may also play a role in cholesterol and fatty acid metabolism. Of the hydroxysteroid 17β‐dehydrogenase family of genes the most closely related to *Hsd17b13* is *Hsd17b11*, although it is not specific to hepatocytes and is also expressed in adipocytes (Horiguchi et al., 2008). HFD‐induced obesity also upregulated *Hsd17b11* and *Hsd17b7* hepatic gene expression in C57BL/6J mice and fenretinide treatment decreased their gene expression compared to HFD mice (Figure 2a RNA‐seq mini‐table *Hsd17b* family of genes). HFD also upregulated *Hsd17b4* and *Hsd17b12* hepatic gene expression in C57BL/6J mice; however, fenretinide treatment had no effect on decreasing these genes. *Hsd17b13* has been linked to MASLD and was one of the top 10% most upregulated genes in C57BL/6J HFD mice, increasing 1.5‐fold (Figure 2a, b). Since HSD17B13 has been reported to have dehydrogenase activity towards retinol (Ma et al., 2019), and *Hsd17b13* was one of the most repressed genes with retinoid treatment, together with the human gene variant data on *Hsd17b13*, this warranted further examination of this hepatocyte‐specific 17β‐HSD family member.

HSD17B13 hepatic protein expression was also induced by a similar amount in HFD mice compared to lean controls (Figure 2c). Fenretinide treatment was able to prevent this induction, with both gene and protein expression levels close to lean C57BL/6J control mice (Figure 2a, c). Fenretinide treatment also repressed *Hsd17b13* expression in parallel with the prevention of liver steatosis in the LDLR^−/−^ mice fed‐HFD plus high cholesterol diet model of dyslipidaemia (Figure 2d) (Thompson et al., 2023). *Hsd17b13* gene expression levels were induced in two more genetic models of metabolic disease and steatotic liver, in leptin‐deficient obese *ob/ob* mice (Figure 2e) and lipodystrophic Seipin/*Bscl2* ^−/−^ mice (Figure 2f) (Mcilroy, George et al., 2018). Fenretinide treatment could not inhibit the increase in *Hsd17b13* obese *ob/ob* mice but gene therapy to restore white adipose tissue (WAT) in *Bscl2* ^−/−^ mice inhibited MASLD and *Hsd17b13* gene expression (Figure 2f) (Sommer et al., 2022). These data taken together with the human gene variant data suggest a role of 17β‐hydroxysteroid dehydrogenases in liver lipid metabolism and an important role for Hsd17b13 in MASLD (Abul‐Husn et al., 2018; Chella Krishnan et al., 2018; Ma et al., 2019).

### 3.3 RNAi‐mediated *Hsd17b13* knockdown in HFD‐induced obesity and MASLD does not affect body weight, adiposity or glucose homeostasis

This RNAi‐mediated knockdown translational strategy has been taken by multiple pharma/biotech companies in ongoing clinical trials (Amangurbanova et al., 2023). Candidate short hairpin RNAs (shRNA) were tested for knockdown efficiency in cells overexpressing mouse *Hsd17b13*, and the most potent (hereafter renamed *shHsd17b13*) was selected for treatment in vivo. To evaluate the role of HSD17B13 in driving steatotic liver, we proceeded to directly knock down the elevated levels of this protein in adult mice with HFD‐induced MASLD thereby circumventing the lack of a protective effect of *Hsd17b13* whole‐body, total genetic knockout from birth in mice. We did not utilise a specific diet (e.g. GAN diet or choline‐deficient HFD) to induce fibrosis or inflammation since these tend to suppress weight gain and adiposity, the primary drivers of MASLD (Loft et al., 2021; Luukkonen et al., 2023; Vacca et al., 2024). Moreover, we had already determined the reciprocal regulation of *Hsd17b13* expression was associated with the simple HFD‐induced obesity beneficial effects of fenretinide (Figures 1 and 2).

**FIGURE 3:**
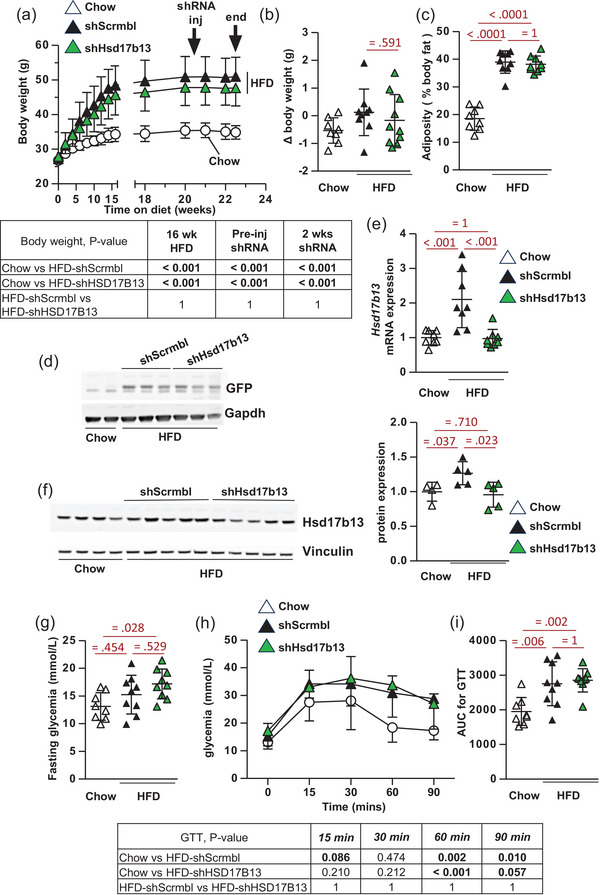
Effect of RNAi‐mediated *Hsd17b13* knockdown on HFD‐induced obesity and glucose homeostasis. (a) Body weights of C57BL/6J mice on HFD (45% kCal fat) or control CHOW diet for 23 weeks. At 21 weeks on HFD, obese mice were randomised for administration of AAV8‐GFP‐*shHsd17b13* or scrambled control shRNA (*shScrmbl*); *n* = 8–9 per group. Change in body weight from 21 to 23 weeks diet (b) and adiposity (% body fat) at week 22 HFD (c) in response to administration of AAV8‐GFP‐shRNA. (d) At 23 weeks, following administration of AAV8‐GFP‐*shHsd17b13* or *shScrmbl*, western blot of GFP in representative mouse liver tissues. (e) *Hsd17b13* gene expression in mouse livers, *n* = 8 per group. (f) Western blot of Hsd17b13 protein expression and vinculin (loading control) in mouse livers and quantification; *n* = 4–5 per group. (g) Fasting (5 h) glycaemia (basal glycaemia from GTT). (h) GTT performed in week 2 post‐treatment with *shHsd17b13* or *shScrmbl*; *n* = 8–9 per group. (i) AUC from GTT for each individual mouse. All data presented as individual data points, means ± SD, and analysed by one‐way ANOVA followed by Bonferroni multiple comparison tests with exact *P*‐values for *post hoc* comparisons between groups (in red or in a table for body weight (a) and GTT (h)). In addition, body weight (a) and GTT (h) have numerous individual data points (*n* > 30), and thus the entire raw dataset is available in Supporting information. In addition, in western blot quantification (f), one‐way ANOVA *P* ≤ 0.05 (HFD‐ *shHsd17b13* vs. HFD‐*shScrmbl*) but required *t*‐test for *P* ≤ 0.05 (HFD‐*shScrmbl* vs. Chow). AUC, area under curve; GTT, glucose tolerance test; HFD, high‐fat diet.

HFD caused increased weight gain and obesity over time compared to chow‐fed C57BL/6J mice, as expected (Figure 3a). At 21 weeks on HFD, obese mice were randomised for administration of adeno‐associated virus (AAV)‐8 constructs encoding either *shHsd17b13* or scrambled control (*shScrmbl*) with the *U6* promoter for high expression of shRNAs. *shHsd17b13* treatment had no effect on body weight or adiposity compared to HFD‐*shScrmbl* control in the following 2 weeks (Figure 3b, c). Hepatic GFP co‐expression confirmed successful AAV8 delivery in HFD mice (Figure 3d). *shHsd17b13* treatment suppressed the elevated *Hsd17b13* expression compared to *shScrmbl* treatment, normalising gene and protein expression to a level similar to that in chow‐fed lean mice (Figure 3e, f). HFD‐induced obesity caused glucose intolerance and *shHsd17b13* knockdown had no effect to alter this compared to *shScrmbl* control mice (Figure 3g–i).

### 3.4 RNAi‐mediated *Hsd17b13* knockdown prevents HFD‐induced MASLD and markers of fibrotic injury

**FIGURE 4:**
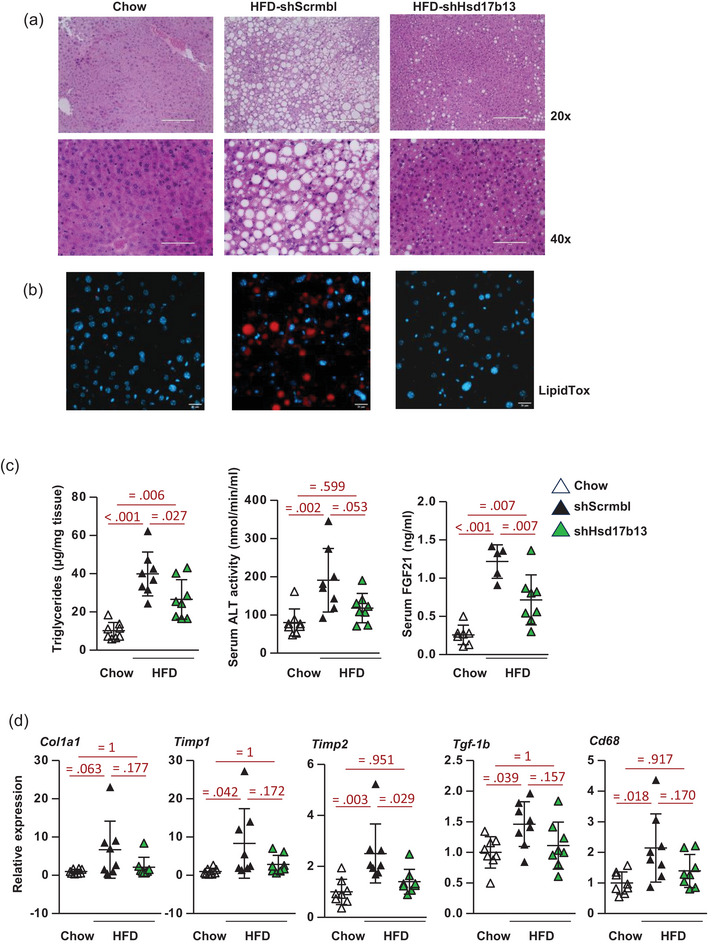
Effect of RNAi‐mediated *Hsd17b13* knockdown on HFD‐induced MASLD. (a, b) H&E staining of representative liver tissues at ×20 and ×40 magnification (a) and fluorescence staining of lipid droplets (b) in C57BL/6J control CHOW diet mice or HFD obese mice treated with *shHsd17b13* or scrambled control (*shScrmbl*). Scale bars are 200 µm (×20) and 100 µm (×40) in H&E images and 20 µm in fluorescence images, respectively. (c) Liver triglyceride levels, serum ALT activity and serum FGF21 levels. (d) Gene expression of fibrosis markers in liver tissue, *Col1a1*, *Col4a1*, tissue inhibitor of metalloproteinases (*Timp*)‐*1* and *Timp2*, *Tgf‐β*‐1 and *Cd*‐*68*. Data in (c, d) are presented as individual data points, means ± SD (*n* = 8 per group), and analysed by one‐way ANOVA followed by Bonferroni multiple comparison tests with exact *P*‐values for *post hoc* comparisons between groups (in red). ALT, alanine aminotransferase; *Cd*, cluster of differentiation; *Col1a1*, collagen type I α1; *Col4a1*, collagen type IV α1; H&E, haematoxylin and eosin; HFD, high‐fat diet; MASLD, metabolic dysfunction‐associated steatotic liver disease; *Tgf‐β*, transforming growth factor β.

HFD‐induced obesity and glucose intolerance led to steatotic liver characterised by increased number and size of lipid droplets as determined by H&E staining of liver tissues and fluorescent staining of lipid droplets (Figure 4a, b). Strikingly, *Hsd17b13* knockdown substantially decreased the number and size of hepatic lipid droplets in HFD mice. In addition, *shHsd17b13* decreased liver triglycerides by 45% from the elevated level in HFD‐*shScrmbl* and back towards a normal level in CHOW mice (Figure 4c first panel). HFD‐induced liver steatosis was associated with elevated levels of serum ALT and serum FGF21 compared to lean mice (Figure 4c second and third panels). *shHsd17b13* decreased both serum ALT levels and serum FGF21 indicating biomarkers of improved liver health compared to HFD‐*shScrmbl* mice.

Persistent excess lipid accumulation and the progression to MASH are associated with the activation of hepatic stellate cells by TGF‐β which leads to the development of fibrosis by collagens and matrix remodelling by tissue inhibitors of matrix metalloproteinases (Friedman et al., 2018). Similar to the RNA‐seq HFD ± fenretinide study (Figure 1 and GEO number GSE220684), 23 weeks of HFD increased the expression of *Col1a1*, *Timp1*, *Timp2* and *Tgf‐β* (Figure 4d). *Hsd17b13* knockdown significantly decreased the expression of *Timp2* and trended to decrease the expression of *Col1a1*, *Col4a1*, *Timp1* and *Tgf‐β* in HFD mice. Similar changes in the pro‐inflammatory macrophage marker *Cd68* were determined. Thus, *Hsd17b13* knockdown and attenuation of excess hepatic triglycerides storage, appeared to inhibit the development of fibrosis and MASH, via suppressing gene expression alterations associated with hepatic stellate cell activation and a pro‐inflammatory environment.

### 3.5 Increasing or decreasing *Hsd17b13* expression does not affect retinoid transcriptional regulator genes or serum RBP4

**FIGURE 5:**
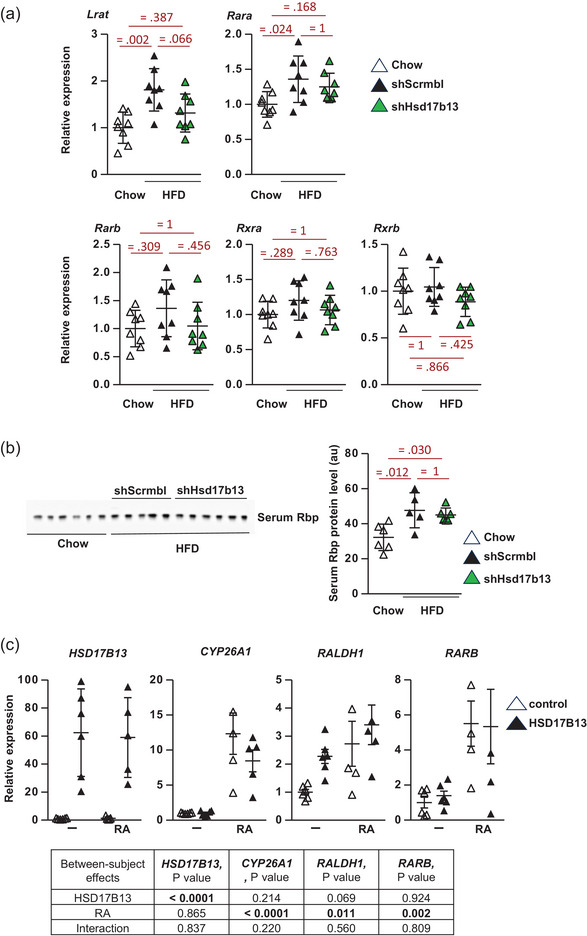
Effect of altered Hsd17b13 levels on retinoid genes and serum Rbp4. (a) Gene expression of hepatic lecithin‐retinol acyltransferase (*Lrat*) and *RA* and *Retinoid X receptors* (*Rar* and *Rxr*) transcription factors (*n* = 8 per group) in C57BL/6J control CHOW diet mice or HFD obese mice treated with *shHsd17b13* or scrambled control (*shScrmbl*). (b) Western blot and quantification of serum Rbp4 levels (*n* = 5–6 per group). Data are presented as individual data points, means ± SD, analysed by one‐way ANOVA followed by Bonferroni multiple comparison tests, with exact *P*‐values (in red) for comparisons between groups. (c) HEK293 cells were transfected with *HSD17B13* or pcDNA3.1 control plasmid to stably express HA‐tagged HSD17B13 and treated with RA (1 µm, 2 h) or DMSO (vehicle control). Gene expression of *HSD17B13* and RA metabolism genes *CYP26A1*, *RALDH1* and *RARB*. Data are presented as individual data points, means ± SD from *n* = 3 biological replicates, and analysed by two‐way ANOVA with exact *P*‐values (in table) for *post hoc* comparisons between groups. RA, retinoic acid.

HSD17B13 has been reported to have in vitro dehydrogenase activity towards retinol and thus modulate RA levels in cells transfected with *HSD17B13* (Ma, Y. et al., 2019). Thus, we determined whether targets of RA signalling were altered with the *Hsd17b13* knockdown. HFD increased hepatic *Lrat* expression, which is associated with altered retinyl ester dynamics in stellate cells and MASH (Figure 5a) (Molenaar et al., 2020; Saeed et al., 2017). *Hsd17b13* knockdown trended to decrease the expression of *Lrat*. *shHsd17b13* had no effect to alter the expression of nuclear receptors *Rara*, *Rxra*, *Rxrb* or *Rarb*, a classic RA target gene (Figure 5a). HFD increased serum Rbp4 levels in *shScrmbl* control mice compared to chow control and *shHsd17b13* treatment had no effect (Figure 5b). Our previous studies showed that the application of the synthetic retinoid fenretinide can strongly induce RA‐responsive nuclear receptor signalling leading to the upregulation of classic RA‐inducible genes (Mcilroy et al., 2013; Morrice et al., 2017). However, our new results suggest that Hsd17b13 does not modulate RA signalling or retinol homeostasis in vivo. To extend these studies further, we examined the effect of *HSD17B13* overexpression and compared it with RA treatment in cells. Although RA could increase the expression of *CYP26A1* and *RARB*, two classic RA‐inducible genes, *HSD17B13* overexpression had no effect alone or in combination with RA to alter their gene expression (Figure 5c). *HSD17B13* overexpression did increase *RALDH1* expression by >2‐fold (*P *< 0.001 by *t*‐test) as did RA treatment; however, there was no additive or synergistic interaction (by two‐way ANOVA). Our results suggest that HSD17B13 does not play a significant role in modulating RA signalling or retinol homeostasis.

### 3.6 Changing Hsd17b13 levels alters hepatic lipid and phospholipid metabolism

**FIGURE 6:**
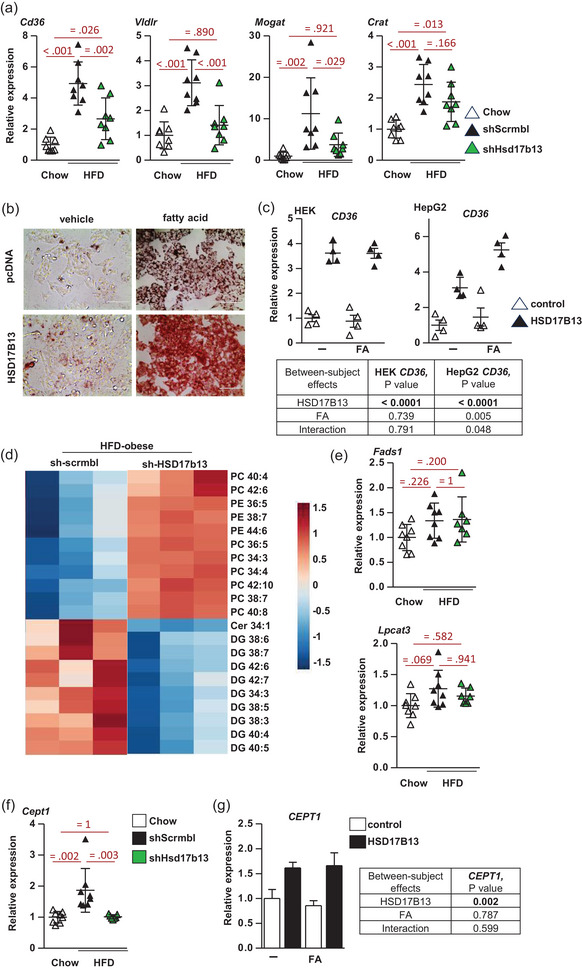
Effect of altered Hsd17b13 levels on lipid and phospholipid metabolism. (a) Gene expression of hepatic lipid metabolism genes (*n* = 8 per group) in C57BL/6J control CHOW diet mice or HFD obese mice treated with *shHsd17b13* or scrambled shRNA (*shScrmbl*). (b) HepG2 cells stably expressing HA‐tagged Hsd17b13 or pcDNA3.1 control plasmid were treated with 1 mM fatty acid (2:1 ratio of oleic and palmitic acid) or methanol (vehicle control). Cells were stained with oil red O. Images are representative of 24 h treatment. Scale bar: 100 µm. (c) Fatty acid translocase *CD36* gene expression in HEK293 and HepG2 cells expressing *HSD17B13* or pcDNA3.1 control and treated with unsaturated/saturated fatty acid mixture (2:1 ratio of oleic acid 0.66 mM and palmitic acid 0.33 mM) or methanol (vehicle control). (d) Lipidomic analysis heatmap of most significant (*P *< 0.05) changes in lipids, enriched in diacylglycerides and phospholipid species in liver tissue from C57BL/6J HFD obese mice treated with *shHsd17b13* or scrambled shRNA (*shScrmbl*); *n* = 3 for each. (e, f) Gene expression of key regulators of phospholipid metabolism (*n* = 8 per group). (g) *CEPT1* (choline–enthanolamine phosphotransferase 1) gene expression in HEK293 cells expressing *HSD17B13* or pcDNA3.1 control and treated with unsaturated/saturated fatty acid mixture (2:1 ratio of 0.66 mM oleic acid and 0.33 mM palmitic acid) or methanol (vehicle control). All data are presented as individual data points, means ± SD. (a, e, f) were analysed by one‐way ANOVA followed by Bonferroni multiple comparison tests with exact *P*‐values for *post hoc* comparisons between groups. (c, g) were analysed by two‐way ANOVA with exact *P*‐values (in table) for *post hoc* comparisons between groups.

Dysregulation of a network of hepatic genes involved in fatty acid metabolism in response to over‐nutrition contributes to excess hepatic lipid accumulation and thus MASLD. We hypothesised that the *Hsd17b13* knockdown‐mediated decrease in the steatotic liver may be associated with a rescue of this impairment. HFD increased hepatic expression of PPARα target genes *Cd36*, *Vldlr*, *Crat* and *Mogat1* involved in fatty acid transport and metabolism, and *shHsd17b13* treatment inhibited this induction (Figure 6a). To determine if these gene expression changes were due to acute changes in HSD17B13 protein or linked secondarily to decreased hepatic lipid accumulation next, we examined the effect of increased *Hsd17b13* expression in cells. We also treated cells with an oleate‐rich mixture of fatty acids containing a low proportion of palmitic acid (oleate/palmitate, 2:1 ratio) representing a cellular model of steatosis that mimics benign chronic steatosis with low toxic and apoptotic effects (Gomez‐Lechon et al., 2007; Nolan & Larter, 2009). Increased *HSD17B13* expression increased triglyceride storage in cells (Figure 6b) and increased *CD36* expression without additional fatty acids in both HepG2 and HEK cells (Figure 6c). The fatty acid treatment further increased triglyceride storage in cells with *Hsd17b13* expression but did not change *CD36* expression. LXRs promote hepatic *de novo* lipogenesis and have been linked with HSD17B13 and MASLD (Su et al., 2017).

Next, we performed a lipidomic analysis of liver tissues to understand the biological function of Hsd17b13 and its role in MASLD. *shHsd17b13* treatment decreased diacylglycerols and increased phospholipids, in particular phosphatidylcholines containing polyunsaturated fatty acids (PUFA) for example, PC 34:3 and PC 42:10 (Figure 6d). We studied the pathway which is responsible for the production of phospholipids and PUFAs. HFD increased the expression of hepatic *Fads1* and *Lpcat3/Mboat5* (*P* = 0.04 by *t*‐test) and *shHsd17b13* treatment did not significantly alter these (Figure 6e). HFD markedly increased hepatic *Cept1* expression and *shHsd17b13* treatment normalised *Cept1* expression in mice (Figure 6f). Choline–ethanolamine phosphotransferase 1 (CEPT1) catalyses the terminal step of the Kennedy pathway and is responsible for PC and phosphatidylethanolamine (PE) production (Gibellini & Smith, 2010). It is also reported to produce the endogenous ligand for PPARα, a transcription factor that is a key regulator of hepatic lipid metabolism genes (Chakravarthy et al., 2009). Increased *HSD17B13* expression in cells increased *CEPT1* expression ± additional fatty acids (Figure 6g). Thus, HSD17B13 appears to be a driver of increased hepatic triglyceride storage and phospholipid remodelling, likely via increased *Cept1* and *Cd36* expression, and may explain at least part of the mechanism of its biological function and role in MASLD.

## 4 DISCUSSION

The multiple overlapping secondary pathologies in response to diet‐induced obesity such as the development of type 2 diabetes, MASLD, atherosclerosis and cardiovascular disease are a global medical burden. Currently, there is only one approved treatment for MASLD or MASH (resmetirom, an oral selective THR‐β agonist) and thus therapies are urgently needed (Sookoian & Pirola, 2024). We have previously reported the beneficial effects of fenretinide to prevent the accumulation of hepatic triglycerides via decreased whole‐body adiposity and associated insulin resistance. Here our novel RNA‐seq study of liver tissue from HFD obese mice ± fenretinide identified major beneficial effects of fenretinide on hepatic gene expression including key drivers of MASLD for example, *Hsd17b13*. Thus, we sought to directly knock down the elevated levels of Hsd17b13 to evaluate its role in driving MASLD and regulation of lipid/phospholipid metabolism.

HSD17B13 gene/protein expression is upregulated in MASLD and transgenic overexpression of Hsd17b13 promotes rapid lipid accumulation in the liver of mice (Su et al., 2014). However, the traditional gene knockout of *Hsd17b13* challenged with several diets to induce steatotic liver and/or fibrosis did not lead to the hypothesised protective phenotype (Ma et al., 2021). Our RNAi therapeutic approach, to suppress *Hsd17b13* levels in adult mice with existing HFD‐induced liver steatosis to decrease hepatic triglyceride storage is a translational approach to investigate the role of Hsd17b13 in MASLD. This RNAi knockdown strategy has been taken by pharma/biotech companies in ongoing clinical trials, for example, Alnylam Pharmaceuticals and Arrowhead Pharmaceuticals (Amangurbanova et al., 2023). Possible explanations for the discrepancy between the phenotype of mouse genetic knockout and the RNAi knockdown approach are if the Hsd17b13 protein affects other proteins in a mechanism independent of its enzymatic activity or total loss of Hsd17b13 activity is compensated for by another protein/pathway.

Su and co‐workers (Su et al., 2022) reported that Hsd17b13 interacts with ATGL on lipid droplets to regulate lipolysis in response to cAMP–protein kinase A (PKA) signalling, a mechanism that resembles a role played by Plin5 (Keenan et al., 2021). Thereby, both Hsd17b13 and Plin5 have been shown to be phosphorylated leading to an increase in ATGL‐mediated lipolysis. While this may be beneficial for MASLD, increased hepatic cAMP–PKA signalling is also associated with excess endogenous glucose production via gluconeogenesis in obesity and type 2 diabetes (Yang & Yang, 2016). Thus, it is not clear if this signalling network can be delineated sufficiently to facilitate targeting for the treatment of metabolic diseases. Recently, the X‐ray crystal structure of HSD17B13 has provided insights into a mechanism for association with the lipid droplet and interaction with ATGL or other potential binding partners (Liu et al., 2023). The findings suggest that HSD17B13 may have an important scaffold function at the lipid droplet.

Distinct changes occur in the liver lipidome with MASLD (McGlinchey et al., 2022; Ooi et al., 2021; Velenosi et al., 2022). Moreover, many genes involved in hepatic PUFA and phospholipid metabolism are important in the formation of biologically important lipids and are linked to metabolic disease by human GWAS and mouse knockout studies, for example, *FADS1*, *ELOVL5*, *LPCAT3*, *MBOAT7*, *CEPT1*, *PLA2G4A*/*cPLA2α*, *PLA2G6*/*iPLA2β*, *PTGDS* and *PTGES* (Jalil et al., 2019; Zhang et al., 2016). The expression of these genes is also altered by dysregulation of a network of transcription factors such as PPARα, LXR and Sterol regulatory element‐binding protein (SREBP). Our lipidomics data suggests that HSD17B13 plays a role in hepatic triglyceride and phospholipid remodelling, whereby knockdown promoted an increase in PCs containing PUFAs, for example, PC 34:3 and PC 42:10. Studies of the human genetic variants of *HSD17B13* (protective against severe liver disease) have reported an increase in phospholipids including PC 34:3 (Luukkonen et al., 2020). *shHsd17b13* knockdown normalised liver *Cept1* expression in mice, and increased *HSD17B13* expression in cells led to increased *Cept1* expression. These results suggest that HSD17B13 may regulate phospholipid metabolism and may explain at least part of the mechanism of its biological function and role in MASLD.

Several human GWAS have identified gene variants in *HSD17B13* which generate loss‐of‐function proteins that associate primarily with protection from the severity of MASLD and fibrosis, that is, progression to MASH (Abul‐Husn et al., 2018; Anstee et al., 2020; Luukkonen et al., 2020; Ma et al., 2019; Zhang et al., 2022). In agreement with our in vivo data, mouse models have shown that inhibition/knockdown of *Hsd17b13* protects against both hepatic triglyceride accumulation and fibrosis (Luukkonen et al., 2023; Su et al., 2022; Wang et al., 2022). It is well known that feeding mice with special research diets leads to a heterogeneous metabolic and liver disease phenotype that is time‐dependent and ranges from simple steatosis to more severe cases of steatohepatitis, fibrosis, and expression of fibrosis and inflammatory markers (Clapper et al., 2013; Loft et al., 2021; Vacca et al., 2024). We did not utilise a specific MASH diet (e.g. GAN diet or choline‐deficient HFD) to induce fibrosis or inflammation since these tend to suppress weight gain and adiposity, the primary drivers of MASLD. Moreover, the reciprocal regulation of *Hsd17b13* expression and fibrosis markers was associated with simple HFD‐induced obesity ± beneficial effects of fenretinide (Figure 1 and 2, GEO number GSE220684).

We determined that *shHsd17b13* knockdown repressed hepatic fatty acid transporter *Cd36* expression in mice, and reciprocally, increased *HSD17B13* expression in cells led to increased *Cd36* expression. Hepatocyte CD36 plays a major role in driving *de novo* lipogenesis and in the development of MASLD (Koonen et al., 2007; Rada et al., 2020; Wilson et al., 2016). Thus, HSD17B13 may play a role in the regulation of key transcription factors (such as PPARa, LXR and SREBP) which coordinate the expression of hepatic lipid homeostasis genes in response to physiological signals.

These data provide strong evidence for the important role of HSD17B13 in driving MASLD via the regulation of hepatic triglyceride storage and phospholipid metabolism. Importantly, this beneficial effect is present without an effect on body weight, adiposity or glucose homeostasis suggesting that directly targeting HSD17B13 is superior to the use of fenretinide, which has diverse mechanisms of action. In addition, treating MASLD may prevent the development of fibrosis/MASH and create an opportunity to clinically treat these without the need to treat co‐morbidities of obesity, type‐2 diabetes and cardiovascular diseases. Thus, the translation potential of targeting HSD17B13 for MASLD/MASH is strong (Amangurbanova et al., 2023; Zhang et al., 2022).

## AUTHOR CONTRIBUTIONS

George D. Mcilroy and Nimesh Mody conceived and designed research; Shehroz Mahmood, Nicola Morrice, Dawn Thompson, Sara Milanizadeh, Sophie Wilson and Philip D. Whitfield performed experiments and analysed data; Justin J. Rochford and Nimesh Mody interpreted results of experiments; Shehroz Mahmood, Sara Milanizadeh, Sophie Wilson, Dawn Thompson and Philip D. Whitfield prepared figures; Shehroz Mahmood drafted the manuscript and Nimesh Mody edited and revised the manuscript. All authors have read and approved the final version of this manuscript and agree to be accountable for all aspects of the work in ensuring that questions related to the accuracy or integrity of any part of the work are appropriately investigated and resolved. All persons designated as authors qualify for authorship, and all those who qualify for authorship are listed.

## CONFLICT OF INTEREST

None declared.

## Supporting information



Body weight and GTT time course, entire raw dataset (related to Fig. 3)

## Data Availability

The data that supports the findings of this study will be made available upon reasonable request. Hepatic RNA‐seq data generated in this study (HFD ± fenretinide, 20 weeks in C57BL/6J mice) has been deposited in the NCBI Gene Expression Omnibus (GEO) database accession number GSE220684.
