# Supplementary material for: Hydroxysteroid 17β‐dehydrogenase 13 (Hsd17b13) knockdown attenuates liver steatosis in high‐fat diet obese mice
Source: Exp Physiol. 2025 Feb 27;110(8):1071–86. doi: 10.1113/EP092535 (PMC12314643; doi:10.1113/EP092535)
Supplement: Supplementary file 1 — Body weight and GTT time course, entire raw dataset (related to Fig. 3) [file EPH-110-1071-s001.pdf]

Age		4	5	6	7	8	9	10
Weeks on HFD		0	0	0	0	0	0	0
group	Ear clip ID	Weight 130120	Weight 160120	Weight 200120	Weight 230120	Weight 300120	Weight 060220	Weight 130220
Chow	1-NM	13	16	22	21	22	24	25
	1-TL	16	18	20	22	24	25	26
	1-TR	14	16	20	22	24	26	27
	1-BR	14	16	18	19	21	23	24
	2-NM	13	16	19	21	23	24	26
	2-TR	14	15	19	23	23	25	28
	2-TL	13	16	20	22	23	25	27
	2-BR	17	20	22	23	24	25	26
HFD	3-NM	13	16	19	20	22	23	25
	3-TR	14	17	20	21	22	24	24
	3-TL	15	19	22	23	25	27	29
	3-BL	11	14	16	17	20	22	24
	4-NM	12	15	18	19	21	23	24
	4-TR	13	16	20	21	25	26	28
	4-TL	14	16	19	19	21	22	24
	4-BR	15	18	21	23	25	26	27
	4-BL	14	17	20	21	24	26	28
	5-NM	18	20	22	23	24	25	26
	5-TR	12	15	19	20	23	24	24
	5-TL	13	16	20	21	23	24	25
	5-BR	14	17	22	23	25	27	28
	5-BL	12	18	22	22	24	26	27
	6-NM	15	17	20	21	22	23	25
	6-TR	14	15	18	20	23	25	27
	6-TL	14	17	19	21	23	24	27
	6-BR	14	18	21	21	23	24	26
	6-BL	15	17	21	22	25	26	28

11	12	13	14	15	16	17	18	19
**Weeks on HFD**	**0**	**1**	**2**	**3**	**4**	**5**	**6**	**7**
**Weight 170220**	**Weight 170220**	**Weight 240220**	**Weight 020320**	**Weight 090320**	**Weight 160320**	**Weight 240320**	**Weight 310320**	**Weight 070420**
	26	26	27	28	29	29.1	29.4	29.9
	27	29	29	30	31	32.2	31.9	31.9
	28	29	30	31	31	32.4	32.5	33.3
	25	27	28	29	30	30.8	31.2	30.9
	26	27	28	29	29	29.3	29.6	29.7
	29	29	30	32	32	32.5	33	32.9
	27	29	29	30	30	30	30.1	30
	26	28	29	30	30	30.6	30.7	31.6
HFD Start	26	30	31	35	36	37.02	38.4	41.3
	25	27	27	30	30	30.1	31.2	32.4
	29	33	34	37	37	38.1	40	39.8
	26	28	28	31	32	33.4	34.8	36.2
	26	27	28	29	30	31	31.9	33.8
	29	32	34	36	38	39.8	41.6	42.9
	25	27	28	30	31	31.3	33.2	34.6
	29	33	35	37	38	38.2	40.1	41.7
	29	32	34	36	37	37.7	39.2	41
	27	31	34	34	34	32.1	33.6	33.3
	27	28	30	32	34	35	36.4	37.3
	27	29	30	33	34	34	36.3	37.9
	30	31	32	34	35	35.2	37.4	37.7
	29	29	29	29	30	30.7	31.8	31.8
	26	28	28	29	31	33.8	35.4	36
	29	31	32	33	34	34.7	36.5	38.5
	27	29	30	31	33	34.3	36	37.2
	27	29	31	33	34	37.6	40.4	41.8
	30	34	39	42	44	47.3	50.6	51.1

20	21	22	23	24	25	26	27	28
8	9	10	11	12	13	14	15	16
Weight 140420	Weight 210420	Weight 280420	Weight 050520	Weight 120520	Weight 190520	Weight 260520	Weight 020620	Weight 090620
30.4	31.1	32.1	32	32.6	32.3	32.6	32.99	33.52
32.4	32.4	32.9	32.6	32.9	32.5	33.3	33.78	33.29
33.5	34.3	34.6	35.2	35.6	35.2	35.8	37.25	36.13
31.2	31.2	31.1	30.9	30.9	31.4	31.7	31.95	32.1
30.3	30.4	30.8	30.9	30.9	31.3	31.6	31.84	31.97
33.5	34.1	34	35.1	35.6	35.8	35.9	36.15	35.68
30.8	30.4	31	31	31	31.6	31.8	32.7	32.15
32.1	32.7	32.9	33.4	34.2	34.8	34.7	35.54	34.93
43.4	43.8	44	44.5	47.7	48.7	49.3	50.27	49.71
32.7	34	35.4	35.1	36.5	37.3	37.3	37.74	37.42
41	41	40.4	40.6	41.3	43.7	48.1	50.58	52.18
37.3	37.6	38.4	37.6	38.6	39.6	40.4	41.04	40.14
35	36.9	37.2	37	39.17	38.7	40.3	41.42	41.1
45	46.1	46.9	47.3	48.53	48.4	49.8	50.64	50.73
36.1	37.5	37.3	37.5	39.65	39.5	42	43.24	43.59
44.1	46	47.3	48.5	50.61	50.6	52.6	52.43	52.57
42.8	43.9	45	45.2	47.25	47.3	49.3	50	50.14
33.5	34.2	34.6	35	36.84	35.9	36.1	36.78	36.42
37.5	38.8	39.2	39	40.84	40.9	41.4	41.36	43.58
40.2	41.5	43.3	45	45.41	45.7	45.9	47.07	47.86
38.7	40.4	40.7	42	43.23	43.8	44.4	46.45	46.73
32.6	33	33.7	34.3	35.86	36.4	36.7	37.92	37.6
37	38	39.5	39.4	41.1	41.9	43.7	44.5	44.4
41	42.1	43.8	45.5	46.65	47.8	48.5	49.6	49.43
38.8	39.8	41.9	42.6	42.65	44.1	45.8	47.73	47.01
44.1	46.8	47.8	47.7	49.25	49.8	50.4	50.3	49.99
52.3	53.6	53.7	53.6	54.1	55.2	56	55.99	55.18

29	30	31	32	33			34	
**17**	**18**	**19**	**20**	**21**			**22**	
**Weight 160620**	**Weight 230620**	**Weight 300620**	**Weight 070720**	**Weight 140720**	**170720**		**Weight 210720**	**Echo weight 240720**
34.37	35.19	35.16	35.7	36.33	Injections (AAV-shRNA)	**Chow**	35.95	36.16
34.14	34	34.39	34.61	35.69			35.21	35.2
37.07	36.96	37.86	37.91	38.55			38.64	39.09
32.69	32.16	32.15	32.2	32.7			33.37	33.66
31.79	31.9	32.81	33.55	33.48			33.35	33.74
36.38	36.56	36.37	37.24	37.11			37.35	37.53
31.91	32.03	31.28	32.27	32.54			32.95	32.22
36.03	35.95	35.87	36.84	36.87			36.96	36.73
51.12	51.72	51.67	51.73	51.52		**HFD-shscrmbl**	51.45	51.33
38.39	38.71	39.32	39.93	41.63			41.5	42.3
53.11	54.31	55.27	55.7	55.61			54.96	55.69
40.82	39.34	40.69	41.13	41.5			41.95	43.07
42.43	43.43	43.66	45.03	45.53		**HFD-sh2**	44.81	45.2
50.9	49.9	50.11	49.8	50.36			51.46	51.26
45.13	45.9	46.77	42.53	47.78			48.39	48.53
53.48	53.87	54.41	55.71	55.64			55.39	56.67
51.02	50.75	51.24	51.39	52.26			52.19	51.83
36.68	36.96	38.02	39.03	39.62		**HFD-sh2**	39.82	40.22
44.79	44.73	45.62	47.51	48.05			47.01	47.46
47.86	48.58	49.67	50.12	50.27			49.79	50.06
47.94	48.48	48.98	49.78	50.46			49.95	51.11
38.66	38.78	39.42	40.7	40.8			41.53	42.17
46	46.71	48.71	48.65	49.39		**HFD-shscrmbl**	49.77	49.72
50.93	52.29	52.2	53.11	53.71			54.37	54.25
49.34	49.91	50.65	51.38	52.3			52.44	52.82
50.99	50.91	51.55	52.72	52.86			52.58	52.14
55.33	56.85	57.13	58.27	59.21			59.17	59.27

	35	
	23	
GTT Weight	Weight 280720	End Weight 310720
35.9	35.32	35.98
35.11	35.13	34.43
37.73	38.75	37.57
32.49	32.98	32.65
32.75	32.53	32.99
36.64	37.07	36.45
32.01	32.72	32.67
36.03	36.09	36.25
50.93	51.2	50.21
42.34	41.62	41.51
55.66	55.34	55.76
42.7	42.5	43.41
48.51	44.54	44.38
50.46	51.11	50.98
46.21	47.38	48.73
55.37	55.42	55.7
44.39	51.46	51.3
39.94	40.16	38.59
46.22	46.47	47.28
49.06	49.73	49.88
49.59	49.8	49.91
41.65	42.13	42.35
49.87	49.25	49.43
54.74	53.34	53.84
52.48	52.33	52.74
52.49	52.26	53.08
58.7	58.7	58.85

Blood Glucose (mg/ml)		Time (mins)				
	ear clip/ID	0	15	30	60	90
Chow	1-TL	14.6	20.3	27.7	18.9	16.4
Chow	2-NM	16.6	29.8	41.3	30.3	22.6
sh2	4-TL	16.6	27.7	42	32.8	27.4
shscr	3-TR	20.9	36.3	42	36.8	42
shscr	6-TL	18.1	34.5	42	42	42
sh2	5-BR	17.9	36	35.5	35.8	33.6
Chow	1-BR	13.2	31.3	14.2	19.2	19.1
Chow	2-TL	11.2	37.1	22.1	14.2	16.2
sh2	4-NM	13.1	36.8	42	31.8	24.1
sh2	4-TR	21.4	39.2	42	40.6	31.2
sh2	4-BL	19.2	28	37.2	30.8	28.6
sh2	5-NM	19.8	31.3	37.5	34.8	25.9
Chow	1-NM	16.2	34.8	34.7	17.9	18.2
shscr	6-NM	13.3	26.1	20.73	16.6	17.3
shscr	6-TR	11.3	32.8	32.1	29.6	27.5
sh2	5-BL	16.9	22.9	17.9	28.9	23.6
sh2	4-BR	14.4	33.5	42	32.8	25.3
Chow	2-TR	20.9	24.6	25.2	13.1	11.4
shscr	3-BL	9.9	37.4	32.5	24.3	15.2
sh2	5-TR	15.2	42	30.7	34.9	23.6
sh2	5-TL	14.7	30.7	31.8	21.2	16
Chow	1-TR	9.9	19.1	42	17.8	19.6
shscr	6-BR	13.7	39.1	26.4	35.9	28.8
shscr	3-NM	15.9	28.2	42	29	28.7
shscr	6-BL	16.6	31.2	28.5	22.7	16.6

**Chow**	**2-BR**	12.3	23.1	17.4	15.7	14.9
**shscr**	**3-TL**	17.4	42	42	42	42

			**15**	**30**	**60**	**90**
t-tests	Chow vs HFD-shScrmbl		**0.0354**	0.1929	**0.0032**	**0.0006**
	Chow vs HFD-shHSD17B13		0.0924	0.0861	**< 0.0001**	**0.0126**
	HFD-shScrmbl vs HFD-shHSD17B13		0.5941	0.6526	0.6454	0.4629
